# Supplementary figures and images for: Connection of core and tail Mediator modules restrains transcription from TFIID-dependent promoters
Source: PLoS Genet. 2021 Aug 12;17(8):e1009529. doi: 10.1371/journal.pgen.1009529 (PMC8384189; doi:10.1371/journal.pgen.1009529)

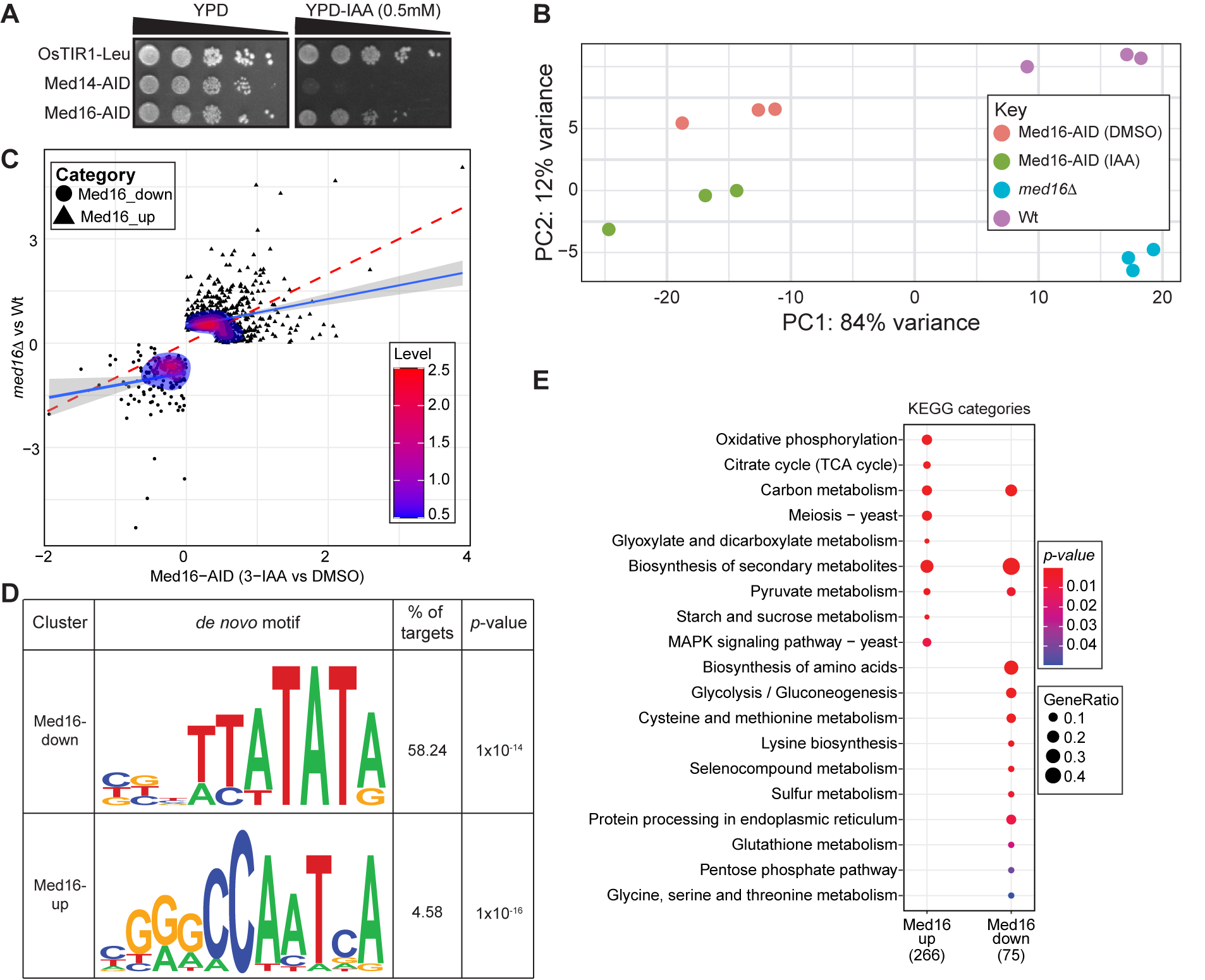

Supplement: S1 Fig — (A) Spot assays assessing growth of parental, Med14-AID, and Med16-AID strains on YPD plates containing DMSO or 500 μM 3-IAA at 30°C. (B) PCA plot of replicate nsRNA-seq experiments performed in WT and med16Δ cells and Med16-AID cells treated with DMSO or 3-IAA. (C) Scatterplot of log2(fold changes) for Med16-regulated genes in WT and med16Δ cells and Med16-AID cells treated with DMSO or 3-IAA with kernel density estimates. (D) Sequence logos of the de novo motifs discovered in the promoters (-400 to +100 bp relative to TSS) of genes in Med16R clusters. (E) Dot plot of KEGG pathways enriched in Med16-up and Med16-down genes. (TIF) [file pgen.1009529.s001.tif]

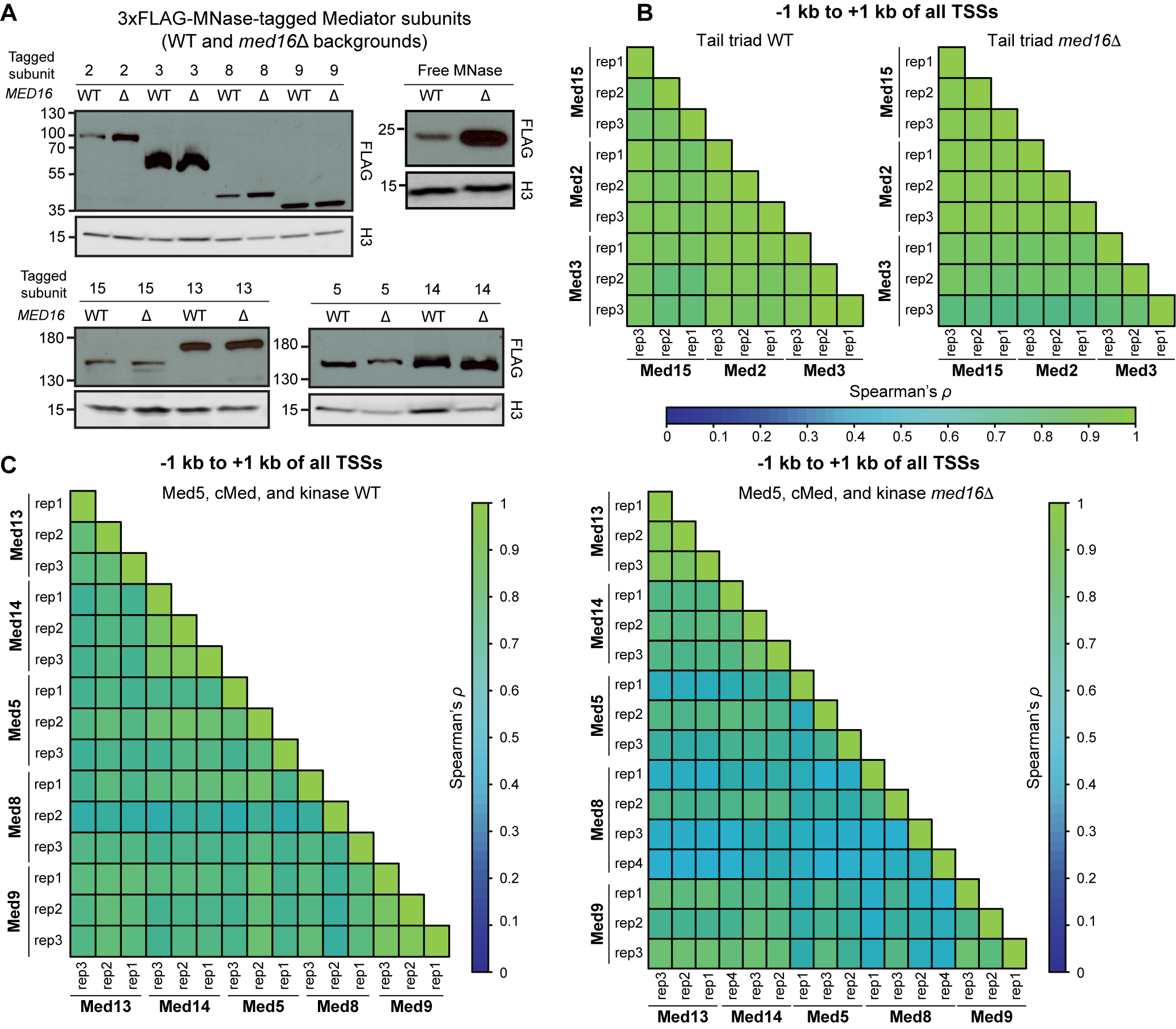

Supplement: S2 Fig — (A) Western blots for 3xFLAG-MNase-tagged Mediator subunits in the WT and med16Δ strains. (B) Correlation matrices of tail triad subunit ChEC-seq replicate signal from the WT and med16Δ strains (-1 kb to +1 kb relative to the TSSs of all genes). (C) Correlation matrices of cMed, kinase, and Med5 ChEC-seq replicates from the WT and med16Δ strains (-1Kb to +1Kb relative to TSS of all genes). (TIF) [file pgen.1009529.s002.tif]

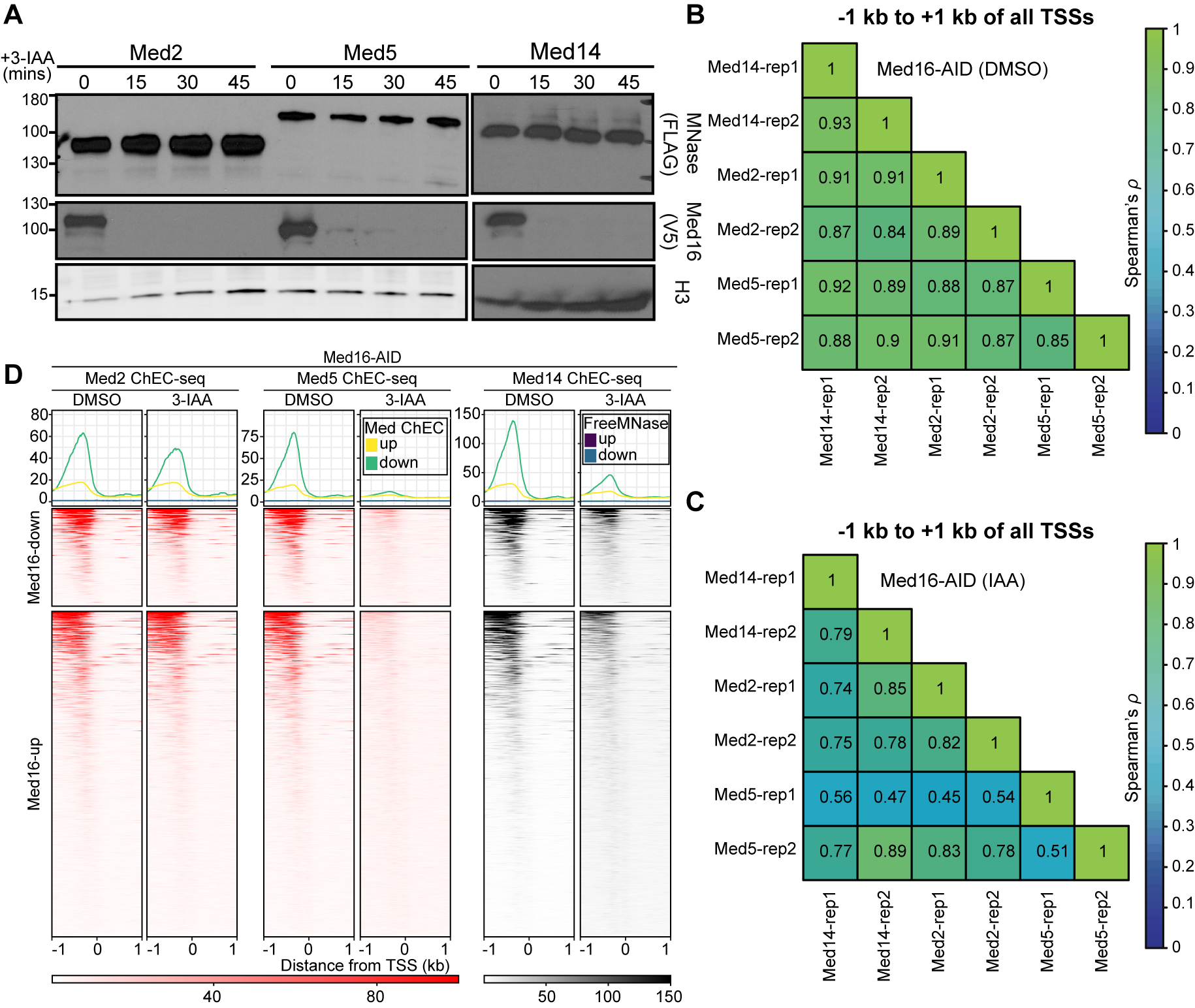

Supplement: S3 Fig — (A) Western blots for 3xFLAG-MNase tagged Mediator subunits after treatment of Med16-AID cells with 3-IAA. (B-C) Correlation matrices of ChEC-seq replicates from DMSO- and 3-IAA-treated Med16-AID cells (-1 kb to +1 kb relative to the TSSs of all genes). (D) Heatmaps of Mediator ChEC-seq signal from Med16-AID cells treated with DMSO or 3-IAA for downregulated and upregulated genes (-1 kb to +1 kb relative to TSSs). (TIF) [file pgen.1009529.s003.tif]

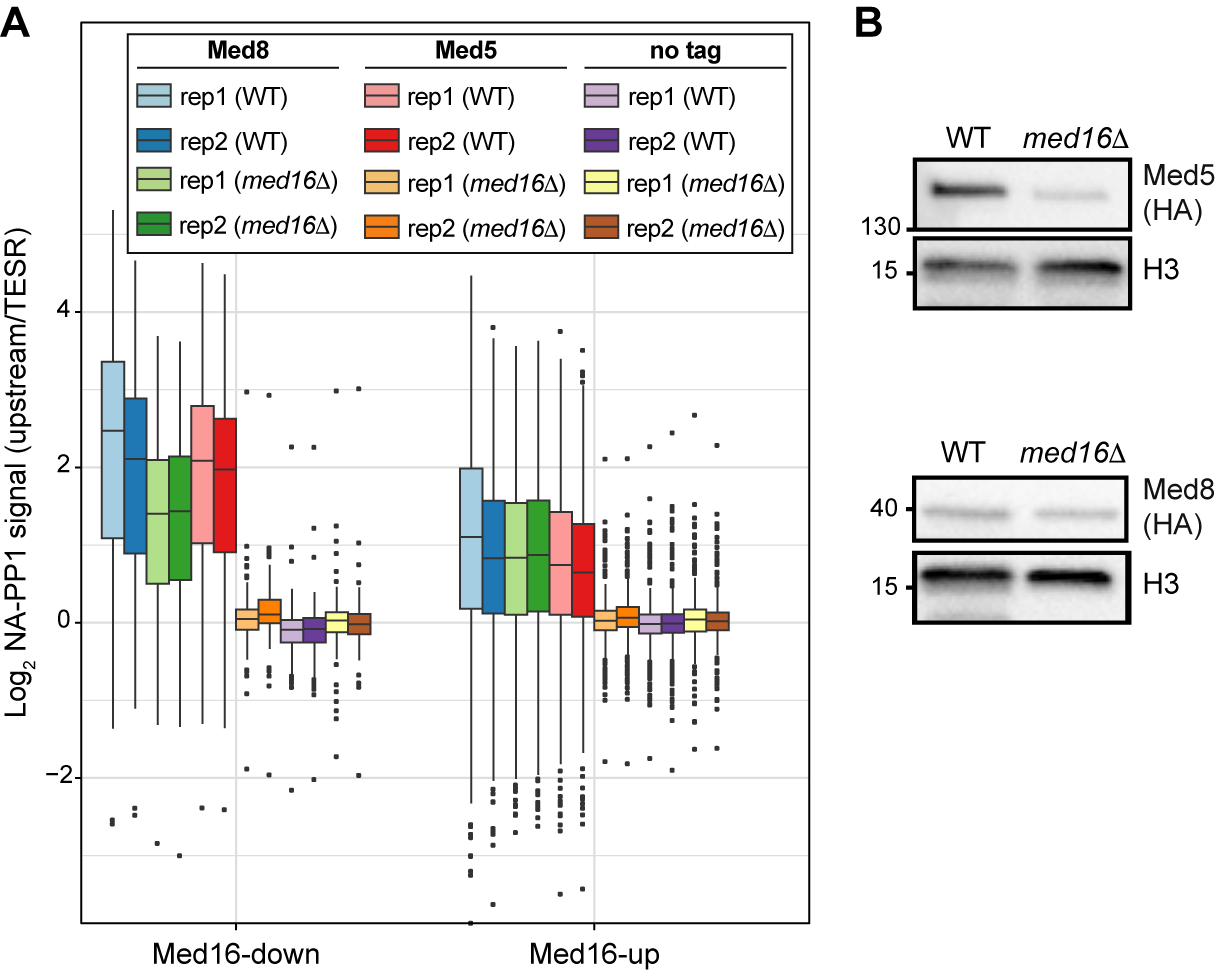

Supplement: S4 Fig — (A) Boxplots of replicate log2 upstream/TESR Med8, Med5, and no-tag ChIP-seq signal from the WT kin28as and med16Δ kin28as strains treated with NA-PP1 for downregulated and upregulated genes. (B) Western blots for 3xHA tagged Mediator subunits in the WT and med16Δ strains. (TIF) [file pgen.1009529.s004.tif]

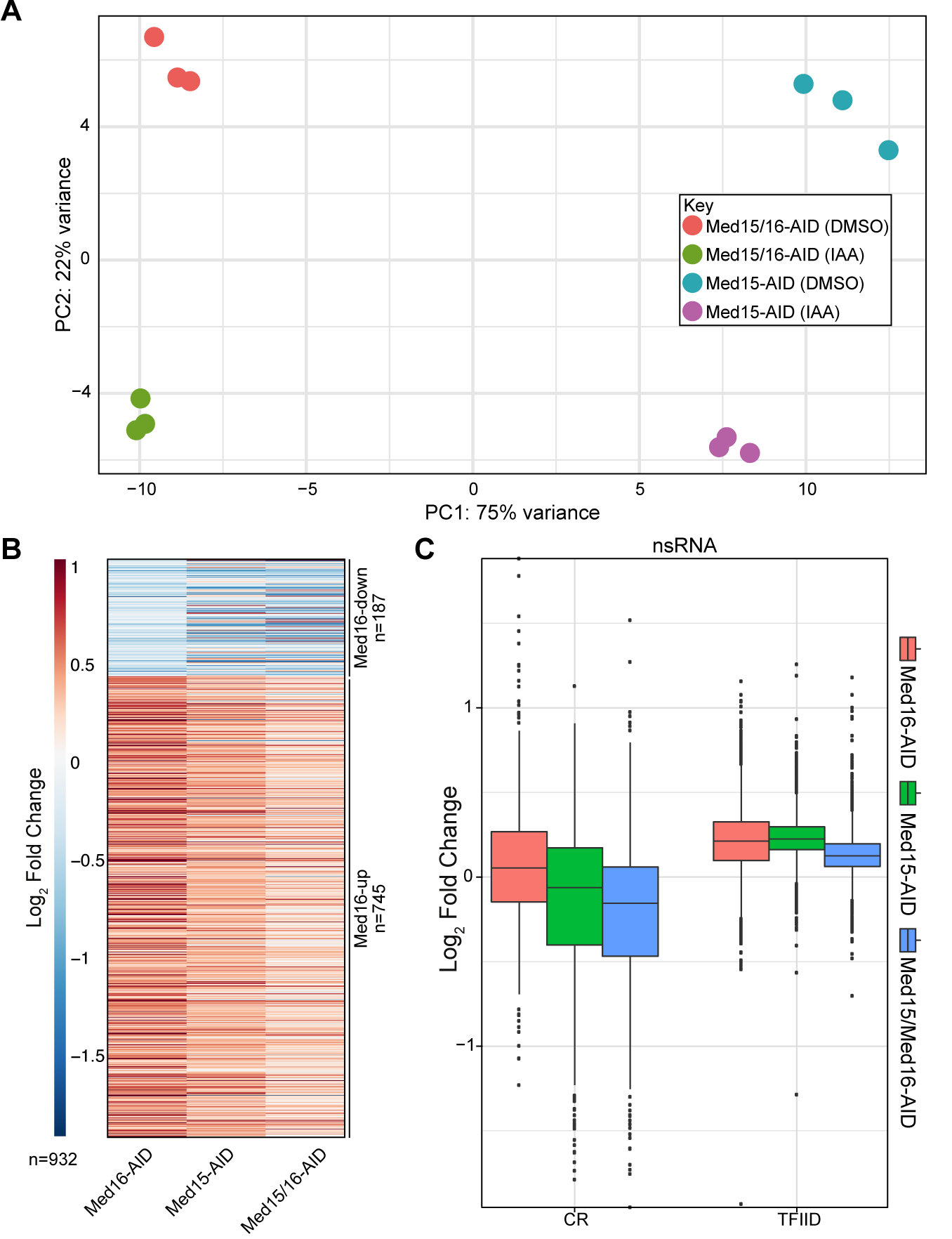

Supplement: S5 Fig — (A) PCA plot of replicate nsRNA-seq experiments performed in Med15/16-AID and Med15-AID cells treated with DMSO or 3-IAA. (B) k-means clustered heatmap (k = 2) of log2 fold changes in nsRNA levels of Med16-regulated gene transcripts in Med16-AID, Med15-AID, and Med15/16-AID cells. (C) Boxplots of log2 fold changes in nsRNA levels of transcripts produced from CR and TFIID genes for the Med16-AID, Med15/16-AID, and Med15-AID 3-IAA versus DMSO comparisons. (TIF) [file pgen.1009529.s005.tif]

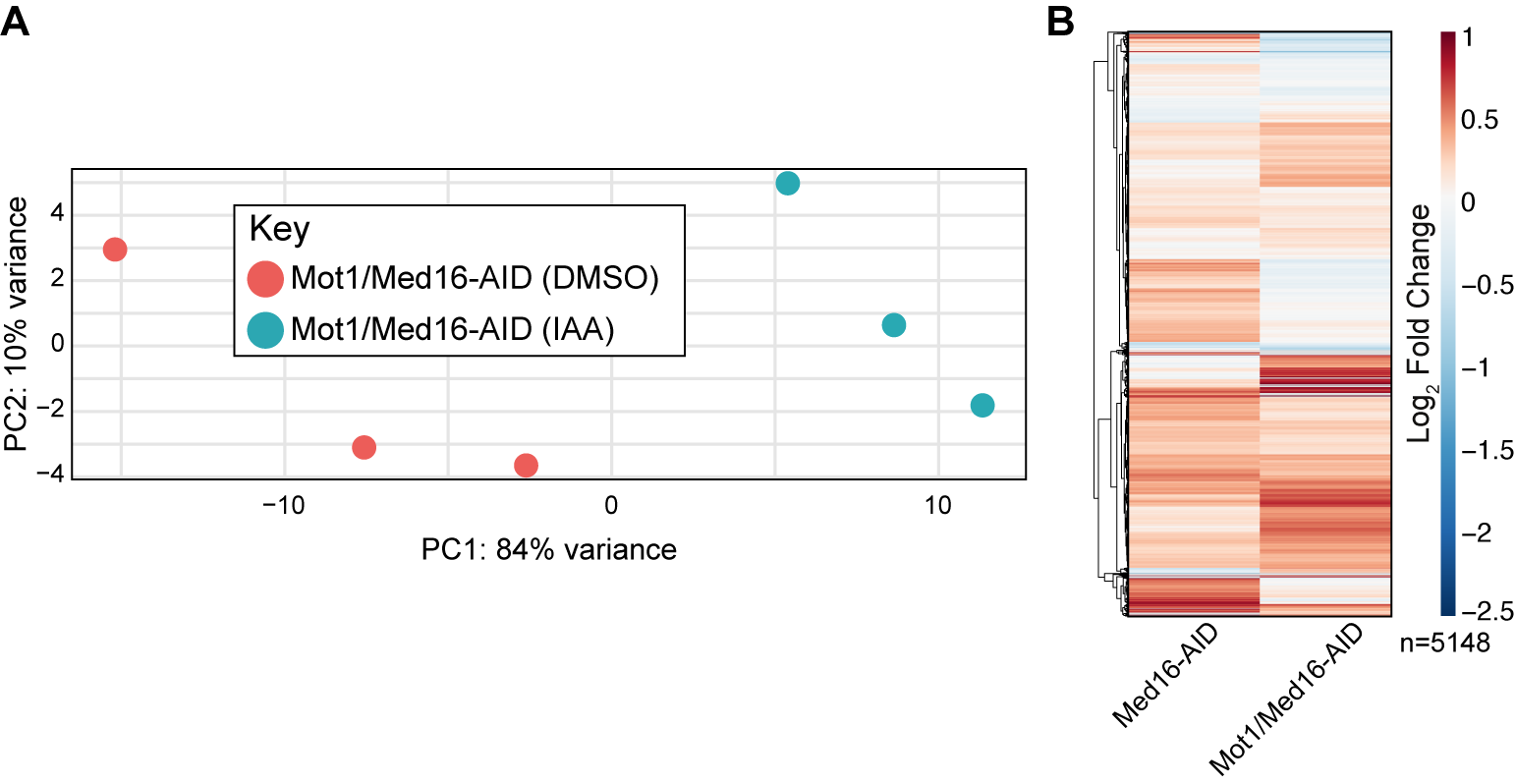

Supplement: S6 Fig — (A) PCA plot of replicate nsRNA-seq experiments performed in Mot1/Med16-AID cells treated with DMSO or 3-IAA. (B) Hierarchically clustered heatmap of log2 fold changes in nsRNA levels of transcripts produced from 5,148 genes encoding verified ORFs for the Med16-AID and Mot1/Med16-AID 3-IAA versus DMSO comparisons. (TIF) [file pgen.1009529.s006.tif]

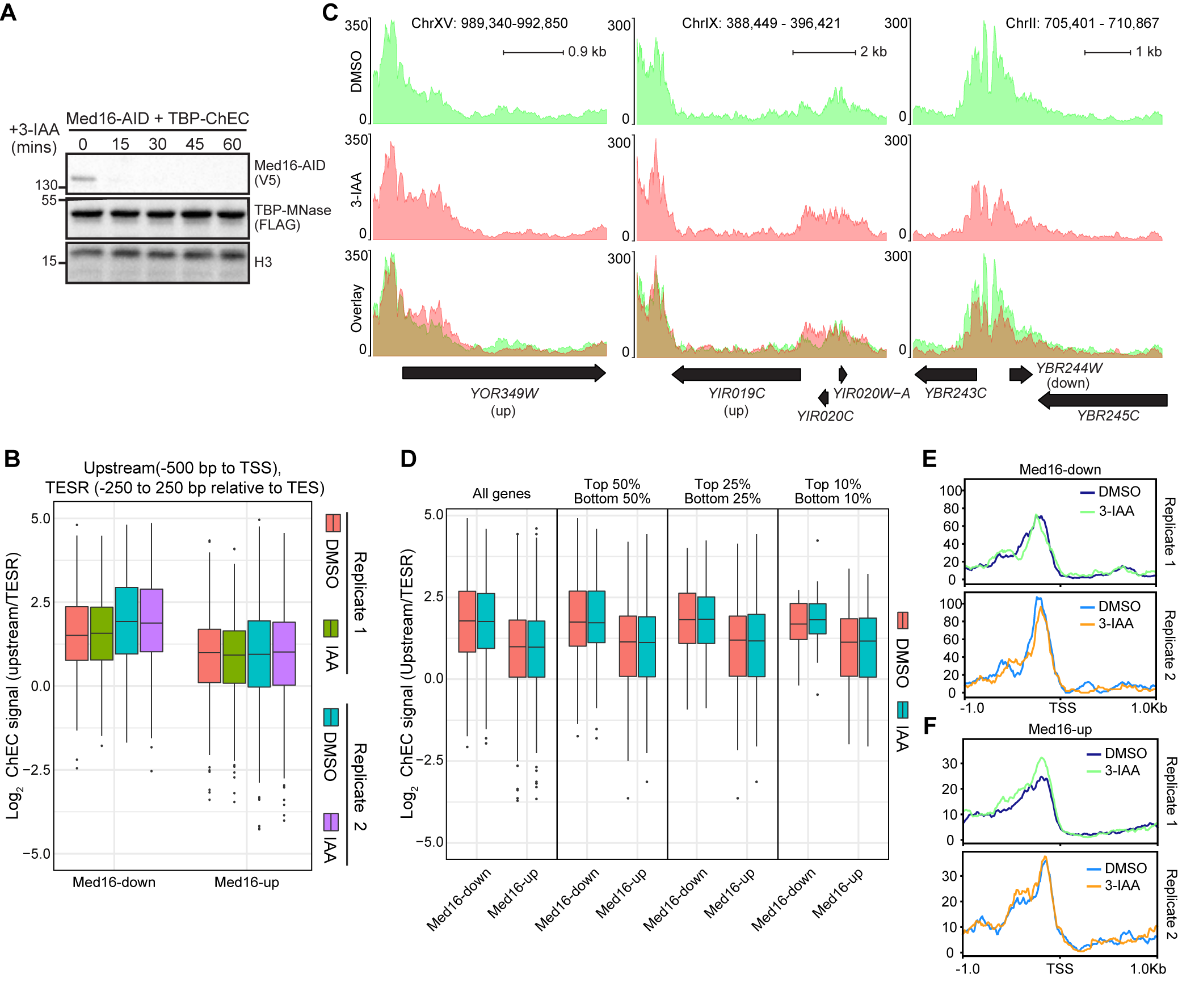

Supplement: S7 Fig — (A) Western blots showing the kinetics of Med16-AID depletion and stability of TBP-3xFLAG-MNase upon 3-IAA treatment of cells. (B) Boxplots of replicate log2 upstream/TESR TBP ChEC-seq signal from Med16-AID cells treated with DMSO or 3-IAA at genes dysregulated by Med16 depletion. (C) Tracks of TBP ChEC-seq signal from cells treated with DMSO or 3-IAA at genes dysregulated by Med16 depletion. (D) Boxplots of average log2 upstream/TESR TBP ChEC-seq signal from Med16-AID cells treated with DMSO or 3-IAA at the given fraction of all genes ranked by nsRNA log2 fold change. (E) Average plots of replicate TBP ChEC-seq signal (≤80 bp fragments) from Med16-AID cells treated with DMSO or 3-IAA at Med16-down genes. (F) Same as (E) but for Med16-up genes. (TIF) [file pgen.1009529.s007.tif]
